# Early Minds: a pilot randomised controlled trial of a mindfulness program in early learning centres

**DOI:** 10.1186/s40814-019-0463-0

**Published:** 2019-06-22

**Authors:** Maya Yaari, Jane Sheehan, Frank Oberklaid, Harriet Hiscock

**Affiliations:** 1 Centre for Community Child Health, Murdoch Children’s Research Institute, Royal Children’s Hospital, 50 Flemington Road, Parkville, Victoria 3052 Australia; 2 Centre for Community Child Health, Royal Children’s Hospital; Health Services Group, Population Health Theme, Murdoch Children’s Research Institute, 50 Flemington Rd, Parkville, Victoria 3052 Australia; 3 Department of Paediatrics, University of Melbourne, Flemington Road, Parkville, Victoria Australia

**Keywords:** Social-emotional learning, Mindfulness, Pre-school, Early education, Wellbeing, Implementation Fidelity

## Abstract

**Background:**

Optimal mental health is critical for a child’s learning and academic functioning. As a universal service, early education centres play an important role in promoting children’s mental health. Social-emotional learning programs are efficacious in reducing behavioural difficulties, enhancing competence, and improving learning abilities. Mindfulness practices, known to promote health and wellbeing in adults, have been adapted to education programs for younger populations, including pre-school children. Despite an increasing use of mindfulness-based programs in pre-school settings, there is a limited number of randomised trials and paucity of data on implementation fidelity of these programs. ‘Early Minds’ is a mindfulness-based program developed by Smiling Mind for 3–5-year-old children. This paper describes a protocol of a pilot randomised control trial, evaluating the implementation of the program in early learning centres (ELCs, i.e. pre-schools) in Melbourne, Australia. The primary aim of this pilot study is to examine the feasibility, acceptability, and fidelity of the program. The secondary aims are to assess the acceptability of the design and measures and to investigate preliminary impacts of the program on child social-emotional outcomes.

**Methods:**

A convenience sample of six ELCs are recruited. Participants include educators, children, and their parents from 3- and/or 4-year-old ELC rooms. Upon completion of baseline surveys, rooms are randomly allocated to intervention and control arms by an independent statistician. ‘Early Minds’ is designed in a flexible delivery manner; meditations and activities are completed at least three times a week. Educators are trained in the program and have access to the activities and meditations on an app. Parents are encouraged to practice with their children at least three times a week. Educators document implementation fidelity throughout the 8 weeks of the program. Parents and educators complete follow-up surveys at 3 and 12 months post-randomisation, capturing feasibility and acceptability, child social-emotional behaviour and sleep, and educator, parent, and family wellbeing outcomes.

**Discussion:**

This pilot study is the first to assess a mindfulness-based program in ELCs in Australia. Data on feasibility and acceptability, implementation fidelity, and potential impact on children’s behaviour will inform the design of adequately powered evaluation trials.

**Trial registration number:**

Australian New Zealand Clinical Trials Registry, ACTRN12618000435280. Date registered 26 March 2018.

## Background

Young children’s mental health has an important role in shaping their learning skills and future outcomes [1–3]. Optimal mental health is achieved not only by eliminating difficulties or symptoms, but also by promoting competence, with optimal mental health being a combination of low difficulties and high competence. Mentally healthy young children are free of not only externalising and internalising symptoms but also succeeding in the cognitive and emotional developmental tasks of their age, gaining skills that lay the foundations for the successful adaptation to future challenges [4, 5]. These include emotion regulation skills—understanding and managing emotions, social skills—getting along with others and empathising, and executive functioning skills—planning, making decisions, and regulating attention [6]. When young children have mental health difficulties and/or low competence to deal with their emotions, behaviours, and relationships, this impairs their ability to learn and function in school. Evidence shows that anything less than the optimal combination of low difficulties and high competence is associated with poorer learning skills and achievements [7, 8]. As a universal service, early learning centres (ELCs) and schools play a key role in developing mentally healthy children, and there is increasing recognition of the importance of acquisition of social-emotional skills in early education contexts [9, 10]. Social-emotional learning (SEL) programs in schools have been shown to improve not only children’s wellbeing but also their academic achievements and learning skills [10–12]. Although all children may benefit from interventions that build their social-emotional skills and executive functioning, evidence suggests that those with higher difficulties at baseline benefit the most [10].

Mindfulness refers to a Buddhist meditation practice that cultivates present moment awareness by paying attention to present experience in the present moment, with openness and without judgement [13]. The ability to direct attention and focus on one thing, and an open, non-judgmental attitude, is developed through the practice of meditation. Mindfulness training has become a common practice in various health settings over the last decades, with robust evidence in clinical adult populations of efficacy in reducing mental health symptoms and improving socio-emotional functioning [14–16] also shown in neural changes in the brain and immune function [17, 18].

More recently, there has been increasing use of mindfulness practices with younger populations. The vast majority of interventions have been conducted with middle and high school students, with some evidence of efficacy for neurocognitive, psychosocial, and psychobiological outcomes [10, 19–21]. Mindfulness programs have recently been extended and adapted to the pre-school age, implemented mostly in early education settings. Results from controlled and non-controlled studies with pre-schoolers show initial promise in reducing externalising symptoms and behavioural problems and hyperactivity [22–25] and in promoting self-regulation skills and executive functions [25–32], prosocial behaviour [22, 28], and language and reading skills [26]. Notably, most studies involving mindfulness practice with children are not randomised control trials and have substantial methodologic limitations, with small sample sizes and potentially biased reported outcomes [19, 21]. Thus, despite some evidence of efficacy, with increasing delivery of programs, there is still a practice-research gap, calling for a stronger evidence base for these programs. Additionally, the literature on programs to improve developmental outcomes and mental health shows that there is considerable variability in the actual delivery of programs in real-life conditions [11, 33]. Implementation fidelity, i.e. the extent to which the program is delivered in the way it was designed, is critical for its efficacy and can also help to understand which elements of the program work for whom and in which contexts. There is a paucity of data on implementation fidelity for mindfulness programs in educational settings [34].

### The Early Minds program

The Smiling Mind Education Program (SMEP) is described by the Smiling Mind organisation as a pre-emptive mental health and wellbeing curriculum, designed to provide accessible and flexible mindfulness educational resources for primary and secondary school teachers, students, and parents. The program includes training modules and a manual for educators, access to the activities and meditations via the Smiling Mind website and app, and a resource guide for parents. The topics covered in the program include awareness, attention, the senses, savouring, movement, recognising emotions, managing emotions, self-compassion, optimism, strengths, gratitude, making decisions, setting goals, empathy, acts of kindness, positive relationships, positive communication, a curious mind, growth mindset, and resilience [35].

An evaluation of an 8-week implementation of the SMEP [36], involving 12 Victorian primary and secondary schools, 1853 students, and 104 teachers, yielded promising results for both teacher and student outcomes. In this controlled trial, at 8 weeks post-intervention, students in SMEP reported a significant increase in their wellbeing and sleep quality, improved sense of safety at school, and reduced classroom disruptions, compared to control group peers. Students reporting greater distress at baseline tended to report greater benefits of the program. Teachers in the SMEP reported improved concentration, sleep, and wellbeing and reduced stress and tension [36].

Based on the success and uptake of the program in primary and secondary schools, Smiling Mind, together with Early Childhood Australia, psychologists and learning experts developed the Early Minds program—a version of the SMEP adapted for pre-schoolers. The adaptation to the pre-school years included simplifying the topics, aligning them with the Australian Early Years Framework [37], and developing short meditations and additional activities that are more suitable and engaging for young children. Two versions of the program exist—one for 3–4-year-old children and one for the 5–6-year-old children. The simplified topics in the Early Minds program include the following: Who am I, Me and My World, At My Best, I love to Learn, and Finding My Voice. Each topic includes one meditation, one mindfulness activity, and two mindful movement activities. Mediation is a formal mindfulness practice, bringing the attention to a point of focus—e.g. focusing on breathing; mindfulness activity is considered an informal practice, through which mindful awareness is brought into daily activities and tasks by engaging all senses and focusing all the attention on the task—e.g. drawing your family while paying attention to the feeling in the hand and the sounds created by using pencil and paper, the colours on the page, and the feeling and thoughts occurring while drawing; mindful movement activities are ‘movement meditations’, which combine elements of formal and informal mindfulness practice—e.g. imitating and learning each other’s or animal movements while becoming aware to the sensations and feelings of the different movements. Smiling Mind and Early Childhood Australia have enlisted the Murdoch Children’s Research Institute (MCRI) to conduct an independent evaluation of the Early Minds program pilot in early learning centres (ELCs) in Melbourne, Victoria.

### Aims and objectives

The primary objective is to evaluate the feasibility, acceptability, and implementation fidelity of the Early Minds program in six Melbourne ELCs. The secondary objectives are to pilot measures of educator and parent-reported child outcomes and investigate preliminary impacts of the program on child, parent, and educator wellbeing and family functioning compared to children and families who do not receive the program. Results of the study may be used to inform the design of a large-scale trial.

### Overall study design and setting

This is a pilot cluster randomised controlled trial, evaluating the implementation of the 8-week Early Minds program by Smiling Mind within ELC rooms compared to standard ELC curriculum. The study involves three points of data collection: baseline and 3- and 12-month post-randomisation follow-up. Parents and educators complete study surveys at baseline, 3 months, and 12 months. Recruitment and training are conducted by Smiling Minds, and the data collection and evaluation are conducted by MCRI. Educator mindfulness-based knowledge, user experience, competence, and actual use of the program inform feasibility, acceptability, and implementation fidelity. Acceptability of the design and measures is assessed by the response rates of educators and parents and completion of the surveys and inform larger trials. Children’s socio-emotional functioning, sleep, and behaviour, reported by parents and educators, and educator and parent wellbeing will indicate potential outcomes of the program.

## Methods and analyses

### Setting

This is a community-based study, set in six ELCs, representing a sample of convenience in the Melbourne metropolitan area. Within each ELC, children are divided into rooms, based largely on their age at the start of the calendar year, i.e. 3-year-old or 4-year-old rooms.

### Participants

Participants are educators within the 3- or 4-year-old rooms at ELCs recruited by Smiling Mind, children who attend the rooms, and their parents. Centres where the majority of families are non-English speaking or with fewer than 15 children per room are not eligible to participate. Children are excluded if their parent’s English language skills preclude their ability to provide informed consent and complete the study surveys, written at a grade 6 reading level.

### Timeline (see Fig. 1).

This study involves three points of data collection: baseline and 3- and 12-month post-randomisation follow-up. Randomisation takes place after baseline data collection.

**Fig. 1:**
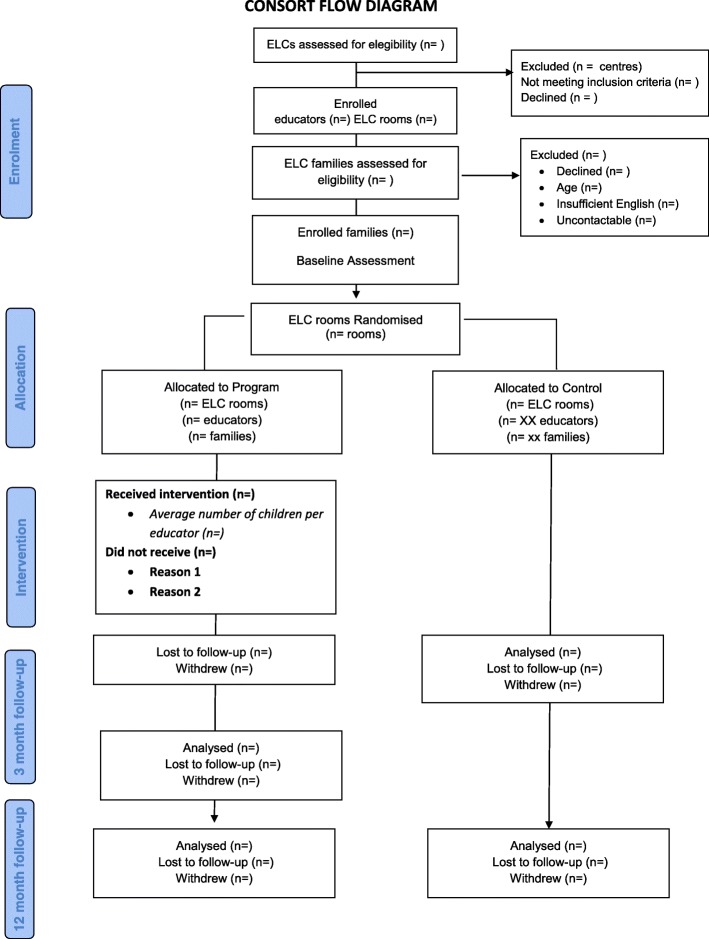
Consort flow diagram

#### ELC recruitment

Recruitment of the ELCs is conducted by Smiling Mind. Following discussions by Smiling Mind with potential ELC Directors regarding the requirements and time commitments involved and assessing eligibility, an agreement is signed with interested centres. Following the ELC recruitment, the research team visits the centre and describes the study procedures to the educators who then sign a Memorandum of Understanding, agreeing to participate in the study. Educators then complete the baseline survey, reporting demographic information and their current experience and knowledge of mindfulness.

#### Child and parent recruitment

All families of children in participating rooms receive information letters, advising that their ELC is participating in the mindfulness evaluation study, that some educators will be trained in the program and others will not, that the trial intervention will go ahead regardless of child/parent participation in the evaluation study, and that the research team will contact the parents to explain more. An ‘opt-out’ approach is used, and parents are asked to return their ‘opt-out’ slip to their ELC if they do not want to be contacted by the study team. If parents do not opt-out within 2 weeks, the centre provides the research team with the family’s contact details and the research team telephone the family to provide further details and assess eligibility. Following this telephone call, interested and eligible parents are sent the study information statement, consent form, and baseline survey via post or email. Upon receipt of the parent survey and consent, the child’s educator is emailed a link to a baseline survey to complete for this child.

#### Randomisation of classes

Upon receiving the completed baseline parent and educator evaluation and child surveys, an independent research assistant randomises ELC rooms to either the program/intervention group or the usual care/control group, stratified by ELC room age (i.e. 3- or 4-year-old room) to ensure younger age rooms are represented in both the program and usual care groups. Children and educators are randomised to the same arm as their room. The independent research assistant conducts the randomisation by selecting a random seed and running a random number sequence using syntax written by an independent statistician. Only educators within the intervention arm are trained in the intervention to reduce contamination in control rooms, particularly if one ELC has an intervention and a control group. Educators and families receive letters to inform them of their group allocation; thus, participants are not blind to allocation. Study staff who are responsible for communication with ELCs and parents are not blind either. However, quantitative analyses will be conducted by research team members who have access only to de-identified data and thus will remain blind to group allocation throughout the analyses.

### Intervention

#### Training

Educators’ training consists of two 1 h online learning modules. The first module provides an introduction to mindfulness and the benefits of regular practising. The second module provides educators with a practical approach to teaching simple mindfulness activities to children aged 3–5 years old. Manuals to support implementation are provided to participating educators as well as access to meditations and activities on the Smiling Mind app.

#### Delivery

The program is delivered by educators over 8 weeks of a school term, at least three times a week. The program includes guided meditations, activities in which mindful attention is practiced in daily activities and mindful movement activities. Activities take 2–15 min each, and the program is designed to be flexible with educator’s choice of activities and how frequently beyond the recommended three sessions per week they are implemented. Parents of children in the intervention arm are provided with a written guide, which includes background information about mindfulness and possible befits of using mindfulness with pre-school age children. Parents are also provided with access to the program meditations and activities on the Smiling Mind app and are encouraged to practice at home with their child at least three times a week.

### Outcomes

Primary outcomes are program feasibility, acceptability as reported by educators and parents, and implementation fidelity as reported by educators. Secondary outcomes are acceptability of the study design and measures by educators and parents, child social-emotional behaviour and sleep as reported by educators and parents, and educator and parent wellbeing.

### Measures (see Table 1)

#### Baseline

Before randomisation, all parents and educators complete a baseline survey including demographic data, their mindfulness knowledge and/or practice (Cognitive and Affective Mindfulness Scale), wellbeing (Depression Anxiety and Stress Scales), and sleep (Longitudinal Study of Australian Children Study). Parents and one educator (who knows the child best) also complete a survey regarding the child’s social-emotional behaviour (Strengths and Difficulties Questionnaire, Affective Reactivity Index, Childhood Executive Functioning Inventory, Approaches to Learning) and sleep (Paediatric Sleep Questionnaire). Parents are also asked about their relationship with the child (PEDS-QL Family Impact Module). Educators are asked about their relationship with children in the room as a proxy of class climate (Student-Teacher Relationship Scale).

**Table 1:** Study measures

Construct	Measures	Time 1 Baseline	Time 2 3 months post-randomisation	Time 3 12 months post-randomisation
Socio-demographic information	Educator: age, number of years as an early childhood educator Parent: family composition, parental education and age, language spoken at home, child age			
Program feasibility, acceptability, and fidelity				
User experience	Study-designed questions assessing use of the program, best and worst aspects of the program, continued use of the program, recommendation of the program to others			
Evaluation of implementation	Study-designed questions assessing usefulness of the program, implementation of the program, barriers to using the program, fidelity to the program, quality of training in the program, comments about the program			
Fidelity	Study-designed questions assessing daily implementation of activities undertaken by educators	daily, for 8 weeks		
Knowledge and use of mindfulness theory and techniques	Study-designed questions assessing previous/current training in and use of mindfulness, techniques, length of time, and frequency that they have used mindfulness techniques			
Parent and educator measures				
Class climate/teacher-student relationships	**Adapted version of the Student–Teacher Relationship Scale short (STRS)** [38, 39]: 15-item validated scale designed to assess the individual teacher-student relationship, but adapted for use with whole class (*α* > .80).			
Dispositional mindfulness	**Cognitive and Affective Mindfulness Scale-revised (CAM-R)** [40]: 12-item validated scale measuring the tendency for the respondent to be mindful in daily life, including four components of mindfulness (attention, present-focus, awareness, and acceptance). Higher scores indicate a more mindful disposition.			
Subjective wellbeing	**3 items used by Smiling Mind to assess self-reported wellbeing of adults using the SM app/website** [41]: Please indicate how each of the following describes your feelings when you think about your life in general? (0 = not at all, 10 = extremely) • How happy do you generally feel? • How content do you generally feel? • How alert do you generally feel?			
Child-related measures				
Child social/emotional behaviour	**Strengths and Difficulties Questionnaire** [42]: 25-item validated measure assessing child’s socio-emotional behaviour, including 5 scales: hyperactivity/inattention, conduct problems, emotional symptoms, peer relationship problems, and prosocial behaviour (*α* = .73).			
Child irritability	**Affective Reactivity Index** [43]: 6-item validated measure assessing child irritability behaviours (e.g. the child gets angry frequently), responses range from 0 (*not true*) to 2 (*certainly true*) (*α* > .80).			
Child sleepiness	**Selected item from the Paediatric Sleep Questionnaire** [44]: Does your/this child have a problem with sleepiness during the day? (yes/no/do not know)			
Child executive function	**Childhood Executive Functioning Inventory (CHEXI)** [45]: 24-item validated measure assessing executive functioning in 4–12-year-old children. It includes 4 subscales: working memory, planning; regulation, and inhibition. Factor analysis for children in kindergarten identifies two factors—working memory (working memory and planning subscales) and inhibition (regulation and inhibition subscales) (*α* > .74).			
Child academic/pre-academic approaches to learning	**Approaches to learning questions**—from the Longitudinal Study of Australian Children **(LSAC)**, adapted from kindergarten through first grade parent social rating scale [46, 47]: 6 items in which parents/educators indicate how frequently the child exhibited the behaviour or characteristic relating to learning experiences. Response scales range from ‘1 = never’ to ‘4 = very often.’			
Child temperament	**Single item from LSAC** [47]: Compared to others I think my child is… *(much easier than average, average, more difficult than average, much more difficult than average, cannot say)*			
Child sleep	**Single item from the LSAC** [47]: How much is your child’s sleeping pattern or habits a problem for you? (*no/small/moderate/large*)			
Family and parent-child outcomes				
Parent mental health	**Depression Anxiety and Stress Scales (DASS-21)** [48]: 21-item validated self-report measure, with three scales for depression, anxiety, and stress. Each scale consisting of 7 items ranging from 1 to 4 rating the extent to which the respondent had experienced each state over the past week (*α* > .85).			
Parent sleep quality	**2 items from Pittsburgh Sleep Quality Questionnaire, modified by LSAC** [47]: • During the past month, how would you rate your own sleep *quantity* overall? • During the past month, how would you rate your own sleep *quality* overall?			
Impact of pre-schoolers behaviour on family relationships and activities	**PEDS-QL Family Impact Module** [49]: This measure includes 5 items assessing problems with family relationships, including communication, stress, conflicts between family members, and difficulty making decisions and solving problems as a family and 3 items assessing problems with daily activities (*α* > .90).			

#### Implementation fidelity

During the 8 weeks of the program, participating educators in the intervention group are asked to complete a daily fidelity checklist. This checklist documents for each activity/meditation the group size (whole group or small group), to what extent it was completed (all, partial), whether children were distressed by the activities (yes/no), and number of times the activity was done that day.

#### Educator program evaluation surveys

At 3- and 12-month post-randomisation, all educators complete an evaluation survey, repeating the baseline assessment of their knowledge and practice of mindfulness, wellbeing, and class climate. Educators in the intervention group are asked about their use and experience of delivering the program.

#### Parent surveys

At 3- and 12-month post-randomisation, all parents complete a survey, repeating the baseline measures of their child’s social-emotional behaviour and sleep, their own mindfulness and wellbeing, and their parent-child relationships. Parents in the intervention group are asked about their use of the program meditations and activities via the Smiling Mind app and their experience of using them.

#### Educator/child surveys

At 3-month post-randomisation, educators from both groups repeat the baseline measures on the child’s social-emotional behaviour and sleep.

Educator and parent surveys are completed online via a web link to Research Electronic Data Capture (REDCap), an online tool, used to build and manage online surveys and databases, which is hosted on a secure MCRI server with restricted access only to researchers involved in the study. Paper version is available if required.

### Sample size

The primary aim of this pilot study is to evaluate the feasibility and acceptability of delivering the Early Minds program within the ELC setting. As such, formal power calculations were not required but a sample of five ELCs with approximately 100 families was deemed sufficient to evaluate the primary aim.

### Analysis plan

Descriptive statistics will be used to collate and report educator and parent responses to open-ended study design questions. Educator’s and parent’s mindfulness-related behaviours change over time; acceptability, frequency of program use, sense of competence, and comments about the program, including challenges and barriers, will be presented.

For quantitative data, the pilot study will use an intention-to-treat analysis. Sample characteristics will be described. Although not a trial designed to determine efficacy of the intervention, mean differences in child outcomes between the intervention and control arms will be reported and will provide important data to calculate sample size and power for any future efficacy trial. For the comparisons of intervention versus control, data will be analysed at the individual level, keeping the nested multilevel structure of the clustered individuals intact using multilevel modelling. This statistical method will be used to account for the correlation of responses within clusters (i.e. classrooms). All estimates will be presented with 95% confidence intervals.

#### Missing data

Where available, we will follow the instructions for each specific outcome measure regarding how to deal with missing data. We will then describe the extent of missing data for the variables in our measures and seek to determine identifiable factors associated with missing data—i.e. was missingness at random or associated with certain characteristics such as family socio-economic status. We are planning to conduct an intention-to-treat analysis as we aim to describe the possible impacts of the program as it would be delivered in ‘real life’ in an early childhood setting and collect data from educators on the types and frequencies of activities. In (hypothetical) cases that an educator was allocated to intervention arm but has not done any activity during the intervention period, we will conduct a complementary per protocol analysis.

## Ethics and dissemination

This study has ethics approval from the Human Research Ethics Committee at The Royal Children’s Hospital, Melbourne (HREC#37267), and the Department of Education and Training Research Committee, Victoria (2018_003686). The study has been registered with the Australian New Zealand Clinical Trials Registry—ACTRN 12618000435280. A report of results of the study in non-technical language will be submitted to Smiling Mind, summaries of the study will be provided to parents and educators, and a copy of the report will be provided to relevant government departments. The results will be published in the peer-reviewed literature and presented to professional audiences at conferences.

### Adverse events

We do not expect to anticipate risks or inconveniences associated with participation in this study. However, in the case an adverse event occurs, the investigators and the ethics committee would be notified and each case would be addressed as required.

## Discussion

Mindfulness practice has demonstrated efficacy in adult and youth populations in improving mental health, by reducing symptoms and improving competence. Despite the increasing use of mindfulness-based programs with children, there is a paucity of empirical data on the efficacy of these programs for pre-school children, as well as data on implementation fidelity. This is a pilot clustered RCT, conducted in collaboration with Smiling Mind, to assess the feasibility, fidelity, and potential efficacy of a mindfulness-based program for 3–5-year-old children in ELCs in Melbourne. The results of this study will assist to build the evidence base for using mindfulness-based programs in ELCs and can inform the design of adequately powered efficacy trials. Within the scope of a pilot and feasibility study, an assessment of children’s outcomes by external, blinded assessors was not feasible and is a limitation in this study. Addition of an objective assessment of executive function, for example, or the classroom environment can provide important additional unbiased evidence in future trials. We will use the data obtained from the study to compare to different indices of minimum clinically important change suggested for the SDQ [50]: difference scores, crossing clinical threshold, reliable change index, and added value scores to inform an a priori decision regarding a measure of clinical significance in a future, fully powered RCT.

## Data Availability

Data will be available upon request.

## Abbreviations

ELC: Early learning centre
MCRI: Murdoch Children’s Research Institute
SEL: Social-emotional learning
SMEP: Smiling Mind Education Program
